# Good to excellent long-term survival of a single-design condylar constrained knee arthroplasty for primary and revision surgery

**DOI:** 10.1007/s00167-021-06636-2

**Published:** 2021-06-14

**Authors:** C. Theil, J. Schwarze, G. Gosheger, L. Poggenpohl, T. Ackmann, B. Moellenbeck, T. Schmidt-Braekling, H. Ahrens

**Affiliations:** Department of Orthopedics and Tumor Orthopedics, Muenster University Hospital, Albert-Schweitzer-Campus 1, 48149 MuensterMuenster, Germany

**Keywords:** Long-term outcome, Condylar constrained TKA, CCK, Revision TKA, Complex primary TKA

## Abstract

**Purpose:**

The purpose of the study was to determine the long-term survivorship, functional outcomes of a single-design condylar constrained (CCK) TKA in primary and revision cases as well as to assess specific risk factors for failure. It was hypothesized that primary CCK TKA had a better survival than revision knees.

**Methods:**

One hundred and forty three patients who underwent revision TKA (*n* = 119) or complex primary TKA (*n* = 24) using a single-design condylar constrained knee system (Genesis CCK, Smith & Nephew) performed at a single institution between 1999 and 2008 were retrospectively included. The median follow-up amounted to 11.8 years (IQR 10.3–14.4). Implant survivorship was analyzed using Kaplan–Meier survival estimates and multivariate Cox regression analysis to identify risk factors for failure. Function was determined using the Oxford Knee Score (OKS).

**Results:**

The implant survival was 86.4% after five, 85.5% after ten and 79.8% at 15 years. A reduced implant survivorship was found in males (HR 5.16, *p* = 0.001), smokers (HR 6.53, *p* = 0.004) and in obese patients (HR 2.26, *p* = 0.095). Patients who underwent primary TKA had a higher revision-free implant survivorship compared to revision TKA at 15 years (100% vs. 76%, *p* = 0.036). The main cause for re-revision was infection in 10% of all revision TKA performed with the CCK design included, while no case was revised for instability.

The median OKS was 39 (IQR 35–44) in 102 patients available for long-term functional outcome.

**Conclusion:**

CCK implants are associated with excellent long-term survival when used in primary TKA; however, survival was worse when used during revision TKA. Males, smokers, obese patients and are at higher risk for revision. While instability and aseptic loosening were rare, infection remains a major concern.

**Level of evidence:**

Level IV, retrospective observational study.

## Introduction

In patients with knee osteoarthritis and instability, severe valgus deformity or bone loss, Condylar Constrained Knee (CCK) implants are widely used, due to their design-related higher stability [5, 12, 27, 29]. Furthermore, in revision knee arthroplasty, the management of instability is considered one of the most difficult issues [13]. In both cases, the CCK represents a compromise between the posterior stabilized (PS) implants, which are mostly used for primary TKA, and rotating-hinge implants. The CCK implants are characterized by an enlarged cam-and-post-mechanism, providing more stability in the sagittal and coronal plane as well as with regards to additional rotational stability [5, 30], even when compared to native knees [1]. However, due to the increased stability, the stress applied on the bone–cement interface is considered to raise the risk of aseptic loosening in these specific implants [4, 20, 23], even when compared to rotating-hinge implants [26]. Additionally, the CCK has a theoretical risk of increased wear of the tibial polyethylene post, potentially leading to instability [3], whereas this could not be ascertained in non-stemmed constrained condylar prostheses when used in primary total knee arthroplasty [6, 7].

Nevertheless, several studies report very good short- to mid-term results, including the use as a primary implant. Furthermore, the clinical outcomes, the range of motion (ROM) and the radiographic outcomes appear to be comparable to PS implants [14, 16, 18, 24, 31] and superior to the hinged constrained implants [28].

Although CCK implants have been available to surgeons for many years [12], there is a scarcity of studies on long-term survival and functional outcome [15, 31].

This study investigates long-term implant survivorship of a single-design CCK implant, analyzes potential risk factors for failure and investigates functional results using the OKS and analysis of range of motion. We hypothesized that overall long-term survival would be greater than 80% and that revision CCK implants had a poorer survival compared to primary CCK implants.

## Methods

Approval of the institutional review board was obtained prior to this investigation (local ethical committee ref. no. 2018-180-f-S). It was conducted according to the principles of the World Medical Association Declaration of Helsinki.

The authors’ institution’s database was retrospectively reviewed and a total of 143 patients who underwent total knee revision arthroplasty (*n* = 119) or complex (including with severe valgus or varus malalignment, major flexion contracture, significant deformity or insufficient collateral ligament stability) primary joint replacement (*n* = 24) using a single-design condylar constrained knee system (Genesis condylar constrained knee (CCK), Smith and Nephew (Smith & Nephew ®, Memphis, TN, USA) between January 1999 and November 2008 were identified. Patients with a minimum follow-up period of 24 months were included. Patients who died or developed complications within 24 months following implantation were included, nonetheless. Follow-up was derived from the last contact with our institution and amounted to a median of 142 months (IQR 123–173) for all patients. Patients who received other (rotating-hinge) knee implants, megaprosthetic reconstructions or patients who had reconstructions following primary or metastatic tumor resection were excluded. Functional assessment was performed using the Oxford Knee Score [21] that measures pain and limitation of function in daily activities as well as measurement of knee range of motion derived from the last clinical examination for all patients with retained implants at latest follow-up compared to preoperative values.

Primary endpoint was defined as prosthetic failure requiring revision and exchange of implant components.

Diagnosis of failure and loosening was based on clinical and radiological findings as proposed by the Knee Society’s evaluation system [9] with three views that examines radiolucent lines around prosthetic components. Joint aspiration was performed in all knees prior to revision surgery and infection was diagnosed using the criteria of the Musculoskeletal Infection Society (MSIS) [22] or prior to 2011 as described by the Center for disease control (CDC) criteria [11]. Treatment success after staged revision in cases of periprosthetic joint infection was defined based on the Delphi consensus criteria [8].

In cases of revision TKA, previous revision surgeries were analyzed and counted. For septic revision surgeries all previous surgeries for PJI were counted including debridement and component exchanges, one-stage revisions and two-stage revisions which then counted as one previous procedure.

### Surgical procedures and implant features

During the course of the study, four different senior arthroplasty surgeons performed the surgeries. A standard medial parapatellar approach was performed in all patients. A tourniquet might have been used by surgeon’s preference if there was relevant bleeding intraoperatively. Systemic intravenous tranexamic acid was not used during the study period. The indications for the use of a CCK prosthesis were mostly made preoperatively on the basis of radiological and clinical examinations. In primary cases with severe valgus or varus malalignment (> 15° in the present study), major flexion contracture (> 25° in the present study), significant deformity (for instance following prior tibial or femoral osteotomy with overcorrection or following tumor curettage or in patients with skeletal dysplasia) or instability, the CCK was used as a primary implant. In a few patients, CCK had to be used due to intraoperative collateral ligament laxity or when instability could not be successfully treated by less constrained prosthesis. In revision cases as default a CCK implant was used, if the collateral ligaments were still intact. If there was the need to reconstruct more extensive bone defects, a rotating-hinge design revision TKA system was used because of the greater modularity available. However, in selected cases with instability only and no major bone loss, the differentiation whether to use a CCK implant or a rotating-hinge design was made by surgeon’s preference. In all cases, existing components were carefully removed, and a thorough debridement was performed removing infected tissue in cases of infection in all compartments of the knee. In all revisions, a minimum of 3–5 microbiological samples were taken and cultured for a minimum of 7–14 days. The general approach regarding the use of cement was to perform a “hybrid” fixation, cementing the femoral shield and the tibial plateau, in combination with a cementless stem whenever possible. However, stems were cemented when diminished bone quality was encountered or if only a short stem anchorage due to ipsilateral hardware or diaphyseal deformity was achievable. The femur and tibia cuts were made using an intramedullary guided cutting block and intramedullary stems for the femur and tibia were used in all cases. In primary and aseptic revision cases gentamicin and clindamycin polymethylmethacrylate (PMMA) bone cement was used (Copal G + C, Heraeus medical, Wehrheim, Germany or Refobacin, Biomet, Warsaw, IN, USA) and in cases of resistant bacteria in septic revisions gentamicin and vancomycin PMMA (Copal G + V, Heraeus medical, Wehrheim, Germany) was utilized. Patients did not receive braces postoperatively and were allowed weight-bearing and active and passive motion as tolerated. All cases of infection underwent at least 2 weeks of tailored intravenous antibiotics, then continuing oral antibiotics for a total of at least 6 weeks in-between stages. Antibiotic suppression was not used. Patients’ characteristics are presented in Tables 1 and 2.

**Table 1:** Patient demographics and surgical details, frequencies

Variable	N	%
Female	91	64
Diabetic	17	12
Smokers	5	4
Primary CC	24	17
Revision CC	119	83
Indication for revision*		
Fracture	4	3
Aseptic loosening	49	41
Instability	30	25
Exchange for PJI	30	25
Malpositioning	7	5

*PJI* periprosthetic joint infection, *CC* condylar constraint

^*^For revisions cases

**Table 2:** Patient demographics and surgical details, metrical data

Variable	Median**	IQR 25–75%
Age at surgery in years	67	62–72
BMI in kg/m^2^	31	25–35
Number of previous exchanges*	1	1–2
length of surgery	126	106–149
Follow-up period in months	142	128–175

^*^For revision cases

^**^There was no significant difference in demographics between patients with primary or revision TKA using the CCK implant

### Statistical analysis

Data collection and statistical analysis were performed using Excel (Microsoft Corporation, Redmont, Washington, USA) and SPSS Statistics for Windows Version 25 (IBM Corporation, Armonk, NY, USA). All patient records were anonymized prior to analysis.

Descriptive statistics were used to analyze distribution of data, means and ranges were calculated for parametric data; medians and interquartile ranges (IQR) for nonparametric data. Survival analysis was performed using the Kaplan–Meier method, differences in survival and influencing factors were assessed using the log-rank test [17] 95% confidence intervals (CI) were calculated. Contingency tables were analyzed using the chi^2^-test. Non-parametric analyses were performed using the Mann–Whitney *U*-Test and the Wilcoxon signed rank test. Hazard ratios (HR) were estimated with their respective 95% confidence intervals (CI) in multivariate Cox regression models.

Statistical significance was defined as *p* ≤ 0.05. A post hoc power analysis (Fisher’s exact-test) for the difference in revision risk at last follow-up between primary TKA and revision TKA was performed using G*Power version 3.1.9.4 [10] resulting in a power of 0.91 with the numbers available.

## Results

### Implant survivorship

**Fig. 1:**
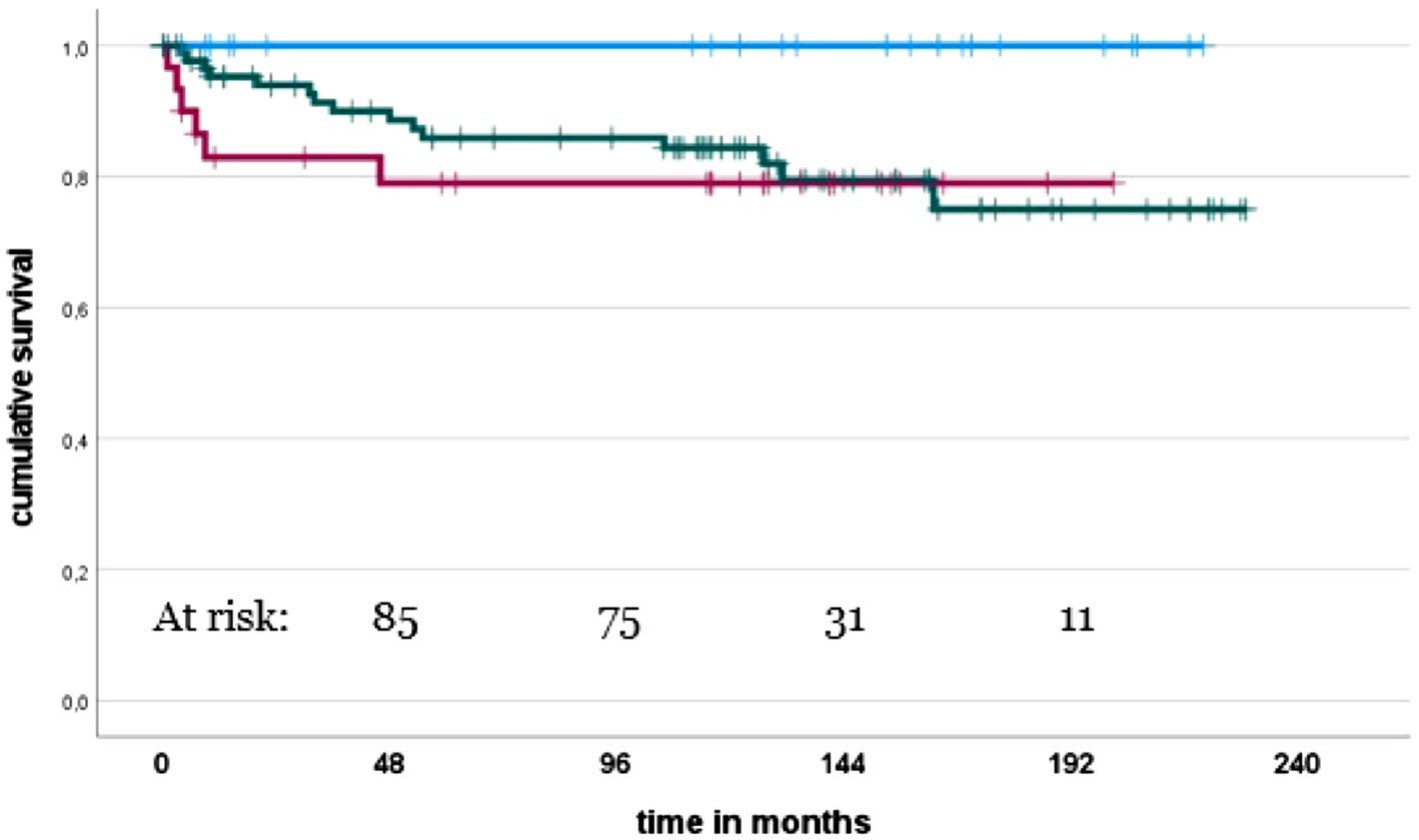
Implant survival with respect to the indication. *Blue line* – complex primary TKA, *Green line* – revision TKA for aseptic failure, *Red line* – revision TKA following PJI (colour figure online)

The cumulative implant survivorship with implant removal for any cause as the primary endpoint was 86.4% after five years (95% CI 80–92), 85.5% after ten years (95% CI 79–91) and 79.8% (95% CI 71–89) at 15 years (Fig. 1).

There was no revision surgery in patients with primary TKA using the CCK design implant.

At the latest follow-up evaluation, 21 patients with a revision TKA using the CCK implant design underwent re-revision with implant removal 17.6% (21/119). Reasons for implant removal were periprosthetic joint infection in 10% of cases (12/119), aseptic loosening in 3% of cases (4/119), non-reconstructable extensor mechanism deficiency requiring arthrodesis in 2% of cases (3/119) and periprosthetic fracture in 1% of cases (2/119). No patient underwent implant exchange for instability in this cohort. Implant failure occurred after a median of 31 months (IQR 6–54).

21 patients had died during the follow-up period of unrelated cause after a median of 132 months (IQR 119–157).

Among patients who were treated for PJI using the CCK implant, the reinfection rate was 13% (4/30). The reinfection-free survivorship in this group amounted to 86% (95% CI 73–99%) after 10 years.

Patients who underwent implantation of a primary CCK had a higher revision-free implant survivorship compared to patients who underwent revision TKA with a CCK at 15 years (100% vs. 75.5% (95% CI 64–86), *p* = 0.036). However, with the numbers available, in revision TKA with a CCK implant, the indication for which the implants was used (aseptic causes, instability, infection) did not influence the revision-free survivorship (n.s.).

### Risk factors for implant failure

A reduced implant survivorship was found in male patients at 15 years (87.8% (95% CI 77–97) vs. 67.1% (95% CI 53–81), *p* = 0.0001) (HR 5.16, 95% CI 1.95–13.67, *p* = 0.001) and in smokers at the time of CCK implantation surgery, (53.3% (95% CI 3–100%) vs. 86.5% (95% CI 80–92), *p* = 0.004 after ten years) which was also found in multivariate analysis (HR 6.53, 95% CI 1.82–23.26, *p* = 0.004).

Furthermore, the number of previous joint revisions was found to be significantly higher in the group with failure of the CCK (median 1 vs. 2, *p* = 0.001).

There was a trend for a diminished implant survivorship in obese patients (BMI > 30 kg/m^2^) at 15 years (70.8% (95% CI 54–86) vs. 86.9% (95% CI 79–95), *p* = n.s.) that was also found in multivariate analysis (HR 2.26, 95% CI 0.87–5.88, *p* = n.s.). On the other hand, there was no significant difference in implant survivorship for patients with diabetes mellitus (HR 0.853, 95% CI 0.261–2.788, n.s.).

On the other hand, age at surgery (n.s.) and the duration of surgery (n.s.) were not found to be significantly associated with implant failure.

### Functional outcome

In 102 patients (71%) functional scores were retrospectively available at last clinical follow-up visits after a median time of 145 months (IQR 126–173). The median OKS was 39 (IQR 35–44).

The median range of motion in extension and flexion improved significantly postoperatively by a median of 10° (80° vs. 90°, *p* = 0.0001).

With the numbers available, there was no association between functional outcome and age (n.s.), obesity (n.s.), gender (n.s.) or between primary and revision cases (n.s.).

## Discussion

The most important finding of the study was that the long-term survival (median 11.8 years) and the functional outcomes of CCK implants are good, particularly when used as a primary implant. However, in males, obese patients, smokers at the time of surgery and patients with more than one prior revision surgery were found to be at increased risk for revision.

A number of studies report on the mid-term outcomes after implanting a CCK prosthesis. Ye et al. reported 92% of good or excellent results after primary TKA and Revision TKA using a second-generation CCK at a mean follow-up of 5.5 years (*n* = 51). In contrast to the findings from the present study, their study found no significant difference between the outcome of the primary and the revision cases [31] with infection being the primary mode of failure. However, there were no instability or aseptic loosening among the complications.

Reina et al. reported on the largest single-design CCK (with a mobile bearing inlay) with a mean follow-up of 4 years. The revision rate for aseptic loosening at 5 years was 3.3% and the overall revision rate for any cause after 5 years was reported to be 9% [25].

However, there are only a few studies on the long-term follow-up of these specific implant type. Cholewinski et al. investigated the long-term results of 43 CCK used as primary implants (NexGen LCCK, Zimmer, Warsaw, IN, USA) [3]. The overall survival of the prostheses at 11 years was 88.5%. Although the complication rate was acceptable at 16% the infection rate was 9.3%, which is considered high in primary arthroplasty. The authors attributed this, among other things, to the selection of the patients, since in this collective 63% had a history of previous surgery and one third of the patients were obese. Based on their overall results, the authors attest that the CCK prostheses have a good long-term survival in primary TKA and state that their data show no evidence for a higher rate for aseptic loosening in this implant type. Even though the survival rates in the present study were lower, there was also a significantly improved revision-free implant survivorship in primary TKA, compared to patients who underwent revision TKA with a CCK at 15 years.

Furthermore, Wilke et al. studied the long-term survival of a single-design implant (TC3 Knee System, DePuy, Warsaw, IN, USA) for revision total knee arthroplasty [30]. The study group consisted of 234 non-septic revision cases with an average follow-up of 9 years. The overall survivorship at 5 years was 91% and 81% at 10 years which is comparable to the present results from this study.

Despite the good long-term survival, several risk factors are currently debated and potential risk factors for further surgery gain importance with longer follow-up. Wilke et al. identified male gender (HR 2.07) as the only variable to increase the risk for revision in a series of 78 patients undergoing revision with a semi-constrained knee design which is in line with the findings presented here that male patients are at higher risk for re-revision (5.16 at 15 years). The authors assume that this may be due to the increased activity level of this group.

Reina et al. demonstrated a higher hazard ratio for aseptic loosening of CCK TKA in young patients and patients with elevated BMI [25]. This finding was explained with the activity level of the patients. However, notably aseptic loosening and instability were no major problems in this series, possibly due to the relatively high patient age at surgery which is often associated with a lower activity level [19]. As the median time to revision surgery was less than three years in the present cohort, it is possible that some patients may have failed due to infection before instability could have become a problem. Future studies should investigate the long-term outcome of CCK implants for specific indications to address this notion.

A recent meta-analysis on varus–valgus constrained primary TKA raised the concern that CCK implants may be associated with significantly increasing revision rates, especially after 5 years [2]. We had no significant increase in revision surgeries after five (86.4%) or ten years (85.5%); however, at 15 years, the cumulative implant survivorship decreased to 79.8%. The results of this meta-analysis on the significantly improved clinical outcomes are comparable to the present results.

While this is a large single-center study that provides long-term results on a single-design implant, its findings must be viewed considering several limitations: one is its retrospective nature that relies on follow-up data. Additionally, while the functional results were collected at long-term follow-up postoperatively, we did not have preoperative results available. However, as studies on long-term function are rare, the present findings can be considered a robust estimate on long-term function when using CCK implants and may be used to guide patients’ long-term expectations.

## Conclusions

CCK implants are associated with excellent long-term survival when used in primary TKA; however, patients who undergo revision surgery using this design are at higher risk for subsequent revision, particularly if other risk factors are present. Patients must be counseled accordingly. While instability and aseptic loosening were rare, infection remains a major concern. These results can give surgeons and the affected patients a better understanding of long-term survival and potential complications associated with the use of CCK implants for patient consultation.
